# Phytochemical Composition and Detection of Novel Bioactives in Anther Callus of *Catharanthus roseus* L.

**DOI:** 10.3390/plants12112186

**Published:** 2023-05-31

**Authors:** Yashika Bansal, A. Mujib, Jyoti Mamgain, Yaser Hassan Dewir, Hail Z. Rihan

**Affiliations:** 1 Cellular Differentiation and Molecular Genetics Section, Department of Botany, Jamia Hamdard, New Delhi 110062, India; yashikab333@gmail.com (Y.B.); jyotimamgain93@gmail.com (J.M.); 2 Plant Production Department, College of Food and Agriculture Sciences, King Saud University, Riyadh 11451, Saudi Arabia; ydewir@ksu.edu.sa; 3 School of Biological and Marine Sciences, Faculty of Science and Engineering, University of Plymouth, Drake Circus PL4 8AA, UK; hail.rihan@plymouth.ac.uk

**Keywords:** anther culture, flow cytometry, GC–MS, phytochemical profiling, ploidy level, secondary metabolites, SEM–EDX

## Abstract

*Catharanthus roseus* L. (G.) Don is the most widely studied plant because of its high pharmacological value. In vitro culture uses various plant parts such as leaves, nodes, internodes and roots for inducing callus and subsequent plant regeneration in *C. roseus*. However, till now, little work has been conducted on anther tissue using plant tissue culture techniques. Therefore, the aim of this work is to establish a protocol for in vitro induction of callus by utilizing anthers as explants in MS (Murashige and Skoog) medium fortified with different concentrations and combinations of PGRs. The best callusing medium contains high α-naphthalene acetic acid (NAA) and low kinetin (Kn) concentrations showing a callusing frequency of 86.6%. SEM–EDX analysis was carried out to compare the elemental distribution on the surfaces of anther and anther-derived calli, and the two were noted to be nearly identical in their elemental composition. Gas chromatography–mass spectrometry (GC–MS) analysis of methanol extracts of anther and anther-derived calli was conducted, which revealed the presence of a wide range of phytocompounds. Some of them are ajmalicine, vindolinine, coronaridine, squalene, pleiocarpamine, stigmasterol, etc. More importantly, about 17 compounds are exclusively present in anther-derived callus (not in anther) of *Catharanthus*. The ploidy status of anther-derived callus was examined via flow cytometry (FCM), and it was estimated to be 0.76 pg, showing the haploid nature of callus. The present work therefore represents an efficient way to produce high-value medicinal compounds from anther callus in a lesser period of time on a larger scale.

## 1. Introduction

*Catharanthus roseus* (L.) G. Don, a member of the Apocynaceae family, is a popular flowering plant. It is an indigenous species to Madagascar and is widely distributed throughout the African, American, Asian and southern European regions. In India, *C. roseus* has been spread across all the major parts of Gujarat, Madhya Pradesh, Assam, Bihar, Uttar Pradesh, Karnataka and Tamil Nadu [1]. The plant is well known for both its ornamental and medicinal value. It produces nearly 130 alkaloids, of which vincristine and vinblastine are the two major compounds that are used in the treatment of leukemia and Hodgkin’s lymphoma [2]. For decades, this plant has been exploited for pharmaceutically active compounds from its native environments and thus is at risk of declining in the wild. Plant tissue culture proves to be an effective biotechnological tool for the rapid propagation of plants under aseptic conditions with a lesser risk of microbial infections [3]. Several in vitro studies using different explants have been successfully conducted for somatic embryogenesis [4] and organogenesis in *C. roseus* [5,6].

In recent times, double haploid (DH) production via anther is a promising option for developing improved plant varieties with high yields of medicinally important bioactive compounds [7]. In vitro anther culture has been attempted in various plants such as *Actinidia arguta* Planch [8] and *Triticum aestivum* L. [9]. Various factors such as stage of anther, culture conditions, plant growth regulators (PGRs) and genotypic and ploidy status determine the success of DH generation [10]. These factors necessitate ascertaining the ploidy status of anther-derived callus to generate true-to-type DH lines, which can be performed with a flow cytometric technique. The flow cytometry method (FCM) measures the genome size by examining the nuclei at a relatively faster rate and thus validates the ploidy levels of different plant tissues [11]. Recent investigations of genome size analysis using FCM have been reported for different plants [12,13]. Phytochemical profiling using gas chromatography coupled with mass spectrometry (GC–MS) has emerged as an important procedure for identifying and quantifying therapeutically significant compounds present in medicinal plants. This technique is relatively faster, accurate and needs a minimum volume of extracts to detect a wide range of bioactive compounds such as alkaloids, long-chain hydrocarbons, steroids, sugars, amino acids and nitro compounds [14]. Major bioactive compounds extracted from different plant parts of *C. roseus* such as stem, root and leaf include vincristine, vinblastine, reserpine, ajmalicine, vindolinine and catharine, which possess anti-cancerous, anti-diabetic, anti-fungal and anti-microbial activities [15]. GC–MS-based profiling has been recently reported for several plants including *Silybum marianum* L. [16] and *Chukrasia velutina* [17], but the information on tissue-culture-raised plants’ phytocompound profiling is relatively much less. The present work, therefore, focuses on investigating the ploidy status of anther-derived callus of *C. roseus* using flow cytometry. The elemental composition of both anther and anther calli was studied using a scanning electron microscopy–energy-dispersive X-ray microanalysis (SEM–EDX) technique. The identification of the bioactive compounds present in methanolic extracts of anther and anther-derived calli was conducted for the first time in *C. roseus* using GC–MS analysis. This report will help to understand and improve the yield of the important pharmaceutical compounds synthesized from anther-derived callus.

## 2. Results

### 2.1. Callus Induction and Proliferation

**Figure 1:**
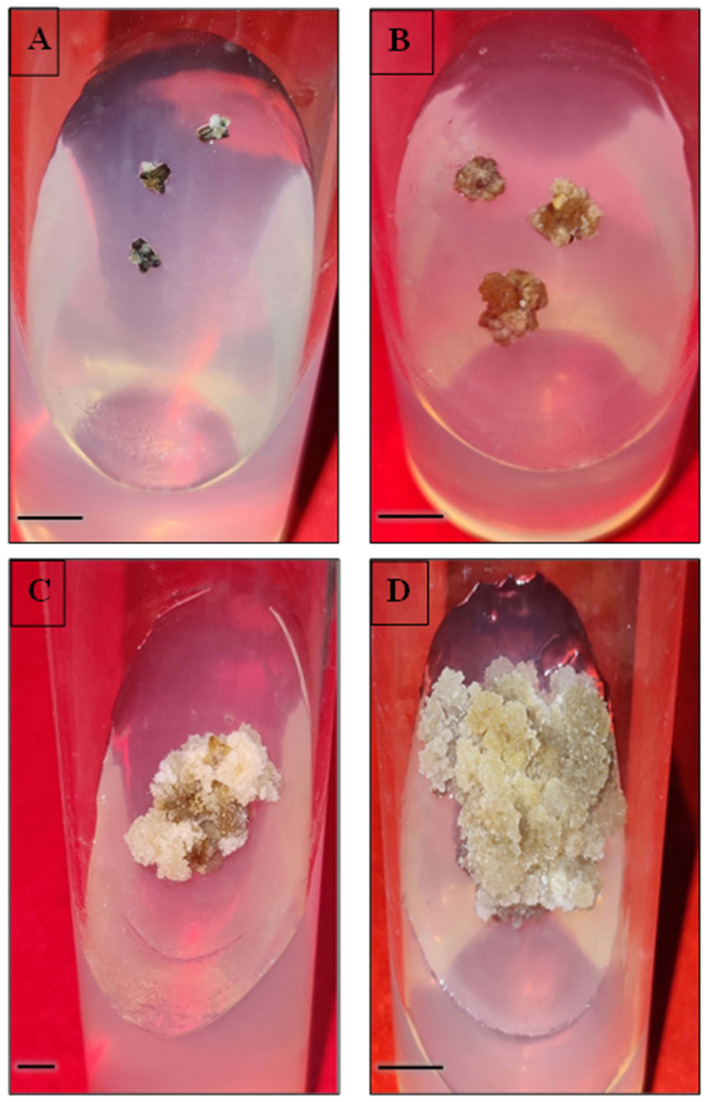

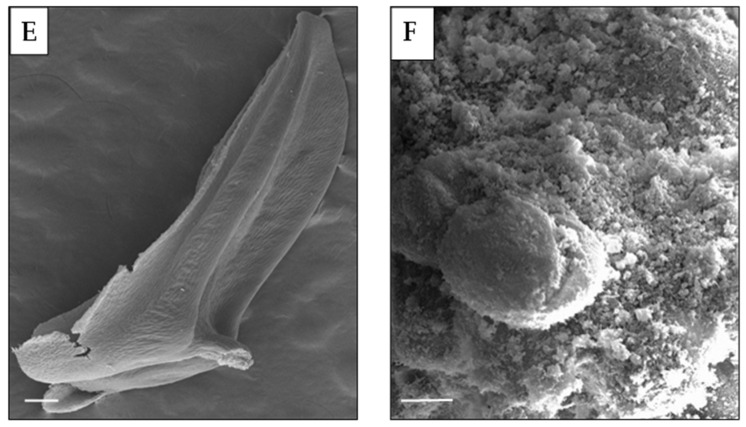
In vitro callus induction, proliferation and scanning electron microscopic (SEM) images of anther and anther-derived callus of *C. roseus*. (**A**,**B**): callus initiation (bars = 0.5 cm); (**C**,**D**): callus proliferation after 6 and 9 weeks, respectively (bars (**C**) = 1.0 cm, (**D**) = 0.5 cm); (**E**): side view of anther (bar = 200 µm); (**F**): a portion of anther-derived callus (bar = 20 µm).

In this study, the anthers were used as explants to induce callus on MS medium augmented with different concentrations and combinations of NAA and kinetin or TDZ alone (Figure 1A). The callusing response ranged from 13.3% to 86.6% on all the tested media (Table 1). Among the PGRs utilized, a combination of NAA and kinetin produced maximum callus (86.6%) at concentrations of 1.0 mg/L and 0.1 mg/L, respectively, followed by 0.75 mg/L TDZ with a frequency of 73.3%. On the other hand, TDZ alone at 0.5 mg/L showed the least incidence of callusing efficiency (13.3%). The highest callus fresh weight was noted to be 1.7 g on MS medium containing 1.0 mg/L NAA and 0.1 mg/L kinetin. The calli obtained were white to pale yellow in color and friable in nature (Figure 1B–D). The anther callus was noted to be recalcitrant, as plant regeneration (embryogenesis and organogenesis) was not achieved on any medium added with various PGR combinations.

### 2.2. Surface Morphology and Elemental Analysis

**Figure 2:**
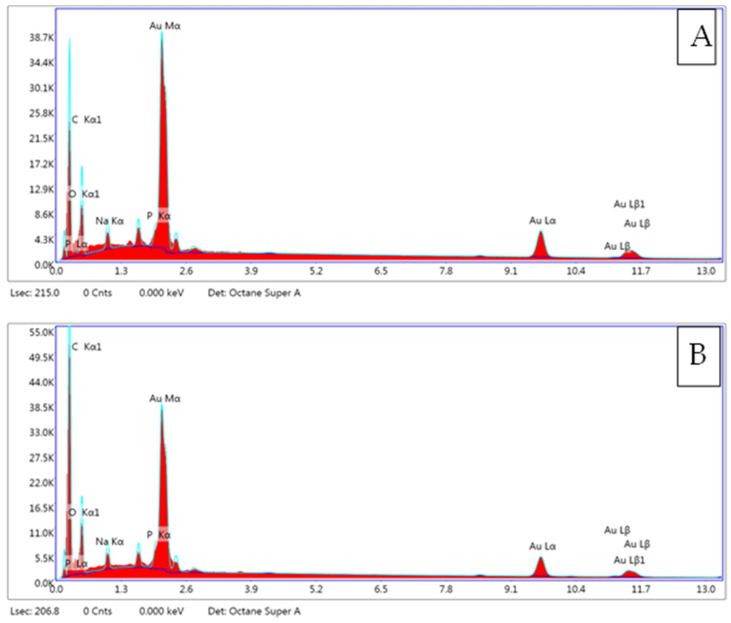
SEM–EDX analysis micrographs showing elemental composition of *C. roseus*. (**A**): field grown anther; (**B**): anther-derived callus.

SEM–EDAX analysis was carried out to determine the elemental composition of anther as well as anther-derived callus. The SEM images and their respective spectra are shown in Figure 1E,F and Figure 2, respectively. The various peaks in both spectra reveal carbon, oxygen, sodium and phosphorous to be the major elements present on the surfaces of anther and anther-derived calli. In both the samples, the carbon and oxygen peaks are prominent and of high intensity, whereas those of sodium and phosphorous are of nearly equal intensity. The quantitative estimation of elements is presented in Table 2.

### 2.3. GC–MS Analysis

**Figure 3:**
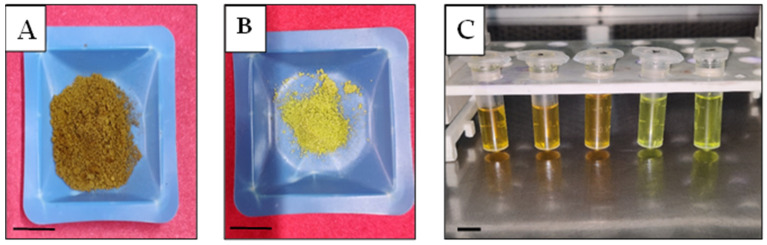
Extract preparation for GC–MS analysis of *C. roseus*. (**A**): dried powder of anther-derived callus; (**B**): dried powder of field-grown anther; (**C**): methanolic extracts of the samples (**A**,**B**).

**Figure 4:**
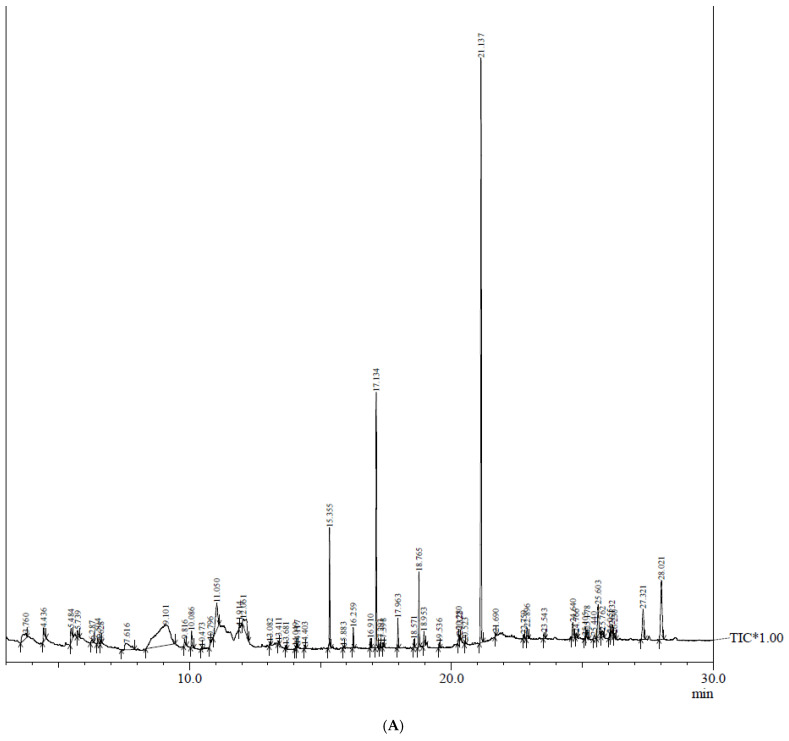

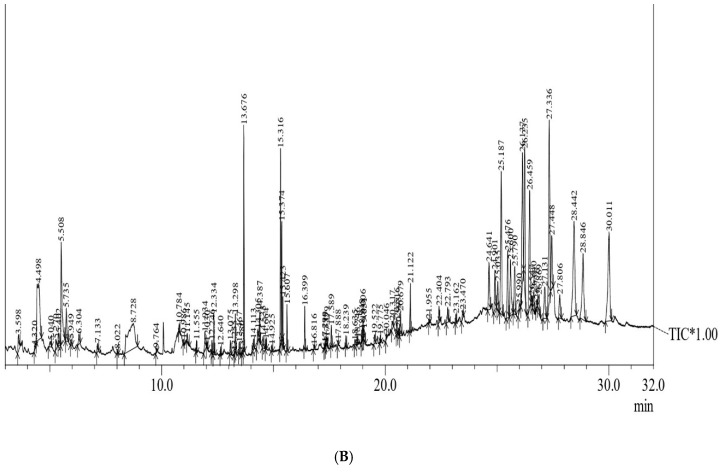
(**A**): GC–MS chromatogram (total ionic chromatogram) of methanolic extract of anthers of *C. roseus*; (**B**): GC–MS chromatogram (total ionic chromatogram) of methanolic extract of anther-derived callus of *C. roseus*.

The bioactive compounds present in methanolic extracts of anthers (donor material) and anther-derived callus of *C. roseus* (Figure 3) were identified using the GC–MS technique. The active principles with their retention time (RT), peak area % (concentration), molecular formula and molecular weight from the NIST library are presented in Table 3 and Table 4, and the GC–MS chromatograms are presented in Figure 4A,B. The chromatograms reveal more than 50 phytocompounds in both methanolic extracts belonging to various classes such as terpenoids, phenols, lignans, steroids, alkaloids and fatty acids.

Among the compounds identified, 1-monoacetin, guanosine, dihydromethyljasmonate, n-hexadecanoic acid, squalene, campesterol, cholestanone and gamma-sitosterol were the most prevalent present in both extracts. Only the methanolic extract of anthers contained bioactives such as cedrol (0.09%), (-)-coronaridine (0.12%), 4-vinylguaiacol (0.14%), vitamin E (0.30%), stigmastanol (0.57%), quinic acid (3.78%) and alpha amyrin (5.54%) (Table 3), and their respective mass spectra are shown in Figure S1A. The extract of anther-derived calli was found to have characteristic metabolites such as pleiocarpamine (0.10%), vindolinine (0.21%), cis-sinapyl alcohol (0.36%), (+)-pericyclivine (0.36%), ajmalicine (0.47%), cycloartenol (0.56%) and beta-stigmasterol (2.09%) (Table 4 and Table 5) having specific mass spectra (Figure S1B).

### 2.4. Flow Cytometric Analysis

**Figure 5:**
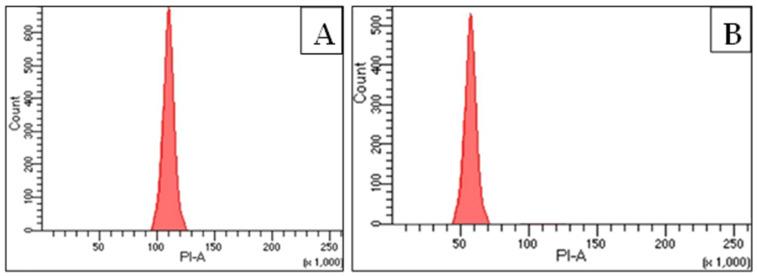
Flow cytometric histograms revealing ploidy level of (**A**) diploid leaves of *C. roseus* (standard) and (**B**) anther-derived callus of *C. roseus*.

The ploidy status of callus obtained from anther was determined using a flow cytometric approach wherein good quality nuclei are a necessity. In this study, the leaves of field-grown *C. roseus* were utilized as an external standard reference (control). The flow cytometric histogram peak of callus reveals that its DNA content was nearly half to that of its diploid counterpart (control) (Figure 5A,B). The nuclear DNA content of anther-derived cell/callus was 0.76 pg compared to the diploid leaves’ DNA (1.51 pg) with a DNA Index (DI) of 0.51 (Table 6). This estimation confirms the haploid DNA status of callus obtained from anther.

## 3. Discussion

The present work was conducted to evaluate the callusing potentiality of anthers of *C. roseus* under in vitro culture conditions. The type and concentration of PGRs used in media strongly affect callusing ability and are different in different plant species. Initially, the anthers were subject to different concentrations and combinations of PGRs amended in MS medium. The results indicate that a high-to-low ratio of auxin: cytokinin concentrations was proven to be the best in inducing callus with a maximum mean fresh weight, which is very similar to Kou et al.’s [19] and Rout et al.’s [20] observations. Likewise, TDZ alone at different concentrations was found to be equally effective in producing callus and subsequent proliferation. Previous reports suggested that TDZ (a cytokinin-like PGR) alone may be used in improving callusing ability in different explants [21,22]. A comparison of the elemental distribution on the surfaces of anther and anther-derived callus was performed using SEM–EDX analysis, revealing a nearly similar elemental composition on both samples. EDX analyzes X-rays emitted from samples receiving a high-energy electron beam. This technique facilitates the qualitative and semi-quantitative detection of surface elements of samples and has been extensively used on various plant species such as sesame [23] and lemongrass [24].

Medicinal plants are an ingenious source of bioactive compounds that fight against several chronic diseases, and these phytocompounds can be identified and quantified using the GC–MS technique [25]. In the current study, phytochemical profiling with GC–MS of methanolic extracts (Figure 5) of anther and anther-derived callus of *C. roseus* has been conducted. The results obtained show the presence of various phytoconstituents, including carbohydrates, alkaloids, phenols, saponins, phytosterols, terpenoids, steroids, etc. A total of 14 bioactives are common in both the extracted samples. However, there are compounds that are exclusive to each sample that confer various biological properties to this plant. The presence of secondary metabolites in callus, which are otherwise not detected in anther tissue, may be due to the fact that certain bioactive compounds accumulate in specific cells or tissues or in a specific growth stage (mostly the stationary phase) of in vitro cultures [26]. Therefore, developing callus from different tissues to obtain therapeutically active compounds is of high significance.

The major compounds of medicinal value present in the methanolic extract of anthers were squalene (triterpene), alpha-amyrin (triterpene), coronaridine (alkaloid) and cedrol (essential oil), which possess anti-oxidant, gastroprotective and hepatoprotective, anti-cancerous and anti-inflammatory properties, respectively [27,28,29,30]. Similarly, in anther-derived calli exclusively, 17 compounds are present having diverse medicinal properties, and these compounds are listed in Table 5. These include stearic acid, linoleic acid, oleic acid, vindolinine, pleiocarpamine, pericyclivine, ajmalicine, 19-epiajmalicine, beta-stigmasterol, cycloartenol, etc. Ajmalicine and vindolinine are well-known alkaloids having anti-cancerous, anti-hypertensive and anti-oxidant properties [15,31]. Recently, an alkaloid named pleiocarpamine has been isolated from the stem bark of *Rauvolfia caffra* and is reported to possess anti-seizure activity [32]. Cycloartenol (a triterpenoid) and stigmasterol (a sterol) have also been detected in present studies and are associated with immunosuppressive, anti-hypercholestrolemic and anti-inflammatory activities, respectively [33,34]. Compounds such as cycloartenol, ajmalicine, vindolinine, pleiocarpamine and pericyclivine have been reported previously in leaf tissues of *C. roseus* [35,36]. Some reports of phytocompounds identified from different tissues using GC–MS were noted earlier [37,38], but till now, no information on the phytocompounds present in anther or anther-derived callus was available for *C. roseus*.

The ploidy status of anther-derived callus was checked using flow cytometry, and the results show that the ploidy of the calli was haploid in nature, confirming the involvement of microspores in developing callus. Similar observations have also been reported for other plant species [7,10,39]. FCM is the widely used approach for determining the ploidy of plants developed through callus, somatic embryos and other in vitro-regenerated pathways [40]. The origin of diploid plants from anthers may be due to the involvement of other somatic cells such as anther wall, filament or flower septum in developing callus. Spontaneous chromosomal doubling can also be a mechanism in the generation of polyploidy in anther-derived regenerants. In certain cases, mixoploids and aneuploids have also been noted in anther cultures of different plants [8,41], but these polyploids were not detected in this experiment. This is the first-ever report of GC–MS analysis of medically significant compounds from anther tissue of *C. roseus*, which enriches the phytocompound library of *Catharanthus* and may be utilized in the pharmaceutical and industrial sectors.

## 4. Materials and Methods

### 4.1. Anther Culture and Growth Conditions

The mature flowers of *C. roseus* were collected from the herbal garden, Jamia Hamdard, New Delhi, and the anthers were used as explants for experimentations. The surface sterilization of flowers was performed following the method of Bansal et al. [3] described earlier. The sterilized anthers were excised from the flowers and aseptically cultured onto agar-solidified basal Murashige and Skoog (MS) medium supplemented with various concentrations and combinations of plant growth regulators (PGRs) and sub-cultured every 3–4 weeks. The cultures were incubated at a temperature of 24 ± 2 °C with 48 μmol/m^2^/s^2^ illumination (white fluorescent light) for a 16 h photoperiod.

### 4.2. Callus Induction and Proliferation

The disinfected anthers were inoculated on MS augmented with different concentrations (alone or in combination) of α-naphthalene acetic acid (NAA), kinetin (Kn) and thidiazuron (TDZ) ranging from 0.1 to 1.0 mg/L for callus induction. Callus formation started within 14–16 days of culture and proliferated on the same medium with successive subculturing. The callus induction frequency and the callus fresh weight were recorded after 6 weeks of culture.

$$\mathrm{Callus}\;\mathrm{induction}\;\mathrm{frequency}\;\left(\%\right)=\frac{\mathrm{Number}\;\mathrm{of}\;\mathrm{explants}\;\mathrm{showing}\;\mathrm{callusing}\;}{\mathrm{Total}\;\mathrm{number}\;\mathrm{of}\;\mathrm{explants}\;\mathrm{inoculated}}\times 100$$

### 4.3. Surface Morphology and Elemental Analysis

The surface morphology and elemental profile of anther and anther-derived callus were determined using energy-dispersive X-ray microanalysis (EDX) combined with scanning electron microscopy (SEM). For this purpose, the samples were primarily fixed with Karnovsky’s fixative and washed with 0.1 M phosphate buffer at 4 °C. Afterward, a series of dehydrations with acetone (30%, 50%, 70%, 90% and 100%) were performed at 15 min intervals, and then critical-point drying was performed at 1100 p.s.i. These samples were then mounted on aluminum stubs and sputter-coated with gold having a 35 nm thick film. Finally, the coated samples were viewed at an accelerating voltage of 20 kV under a scanning electron microscope (Zeiss, Oberkochen, Germany) equipped with EDAX.

### 4.4. Preparation of Extracts

The methanolic extracts of both samples were prepared according to the protocol of Hussain et al. [42]. About 1.0 g of anther and anther callus were shade dried and crushed into fine powder using mortar and pestle (Figure 3A,B). Each sample was then extracted in 5.0 mL methanol in an orbital shaker for 48 h. Afterward, the extracts were filtered through Whatman filter paper no. 1 and evaporated to dryness. The obtained extracts were stored in an airtight container with proper labeling at 4 °C for further use (Figure 3C).

### 4.5. GC–MS Analysis

GC–MS analyses of these extracts were conducted on GC–MS QP-2010 equipment (Shimadzu, Japan) at Advanced Instrumentation Research Facility (AIRF), JNU, New Delhi. The program settings were as follows: Helium was used as a carrier gas (1 mL/min), and the initial and final temperatures were programmed at 100 °C and 260 °C, respectively, with a hold time of 18 min. Ion source temperature was 220 °C with an interface temperature of 270 °C and solvent cut time of 2.5 min. Other specifications included: detector gain mode relative to the tuning result, detector gain +0.00 kV, threshold of 1000, start time 3 min, end time 39.98 min, event time 0.3 s, scan speed of 2000, start *m*/*z* 40.00 and end *m*/*z* 600.00.

### 4.6. Metabolite Data Processing and Analysis

The bioactive compounds were identified using the mass spectral database of the NIST17 library. The unknown compounds’ spectra were compared with the known phytocompound spectra available in the NIST library, and the name, molecular weight and structure of the compounds were determined.

### 4.7. Flow Cytometric Analysis

The ploidy status of anther-derived calli was examined using the flow cytometry method as described by Galbraith [43]. A total of 3 samples of anther-derived callus were randomly chosen, along with a reference standard of diploid leaves of *C. roseus* with a known 2C DNA content of 1.51 pg [44]. Approximately 50 mg of callus was added to a Petri plate having 1.0 mL ice-cold Galbraith’s buffer (nuclei isolation buffer) and finely macerated with the help of a surgical blade. The homogenate was then filtered with a 100 µm nylon mesh to eliminate larger cellular remnants and was finally stained with 50 µg/mL PI RNase (propidium iodide RNase) (Sigma-Aldrich, St. Louis, MO, USA) for 8–10 min. The samples were incubated in the dark at 4 °C for about 40 min and eventually examined on a BD FACS(Calibur) flow cytometer (BD Biosciences, Franklin Lakes, NJ, USA). The relative nuclear DNA of anther-derived callus of *C. roseus* was estimated using the below formula [45]:

$$\mathrm{Nuclear}\;\mathrm{DNA}\;\mathrm{content}\;\mathrm{of}\;\mathrm{sample}\;\left(\mathrm{pg}\right)=2C\;\mathrm{DNA}\;\mathrm{content}\;\mathrm{of}\;\mathrm{standard}\;\left(\mathrm{pg}\right)\times \frac{\mathrm{mean}\;\mathrm{position}\;\mathrm{of}\;G0/G1\;\mathrm{peak}\;\mathrm{of}\;\mathrm{sample}}{\mathrm{mean}\;\mathrm{position}\;\mathrm{of}\;G0/G1\;\mathrm{peak}\;\mathrm{of}\;\mathrm{standard}}$$

### 4.8. Statistical Analysis

In the tissue-culture experiment, three explants (anthers) per culture tube were inoculated with five replicates of every experimental treatment, and each experiment was repeated twice. The data are expressed as mean ± standard error, and the analysis was performed using one-way analysis of variance (ANOVA). The significance of mean difference was determined using Duncan’s multiple range test (DMRT) at *p* < 0.05 using SPSS Ver. 26.0 (SPSS Inc., Chicago, IL, USA) [46]. The flow cytometric study was repeated thrice with randomly chosen standard (donor plant) and callus samples.

## 5. Conclusions

The in vitro culture technology was successfully employed to obtain callus from anther tissue of *C. roseus*, an important medicinal plant. The callus was checked for its ploidy status using flow cytometry and was found to be haploid in nature. The calli obtained from anther were then subjected to GC–MS analysis for phytocompound identification. Among the bioactive compounds identified, ajmalicine, vindolinine, pleiocarpamine, pericyclivine, stigmasterol, campesterol and squalenes were detected and have a wide range of biological activities. From this study, it can then be concluded that anther-derived calli are a potent source for developing new therapeutic drugs with larger-scale applicability in pharmaceutical sectors.

## Figures and Tables

**Table 1:** Effect of different concentrations and combinations of PGRs on callus induction and callus biomass (fresh weight) from anther explants of *C. roseus*.

PGRs	Concentration (mg/L)	Callusing Frequency (%)	Mean Fresh Weight (g)
Control	0	0 ^e^	0 ^c^
NAA + Kn	0.1 + 1.0	26.6 ± 12.4 ^cde^	0.8 ± 0.3 ^abc^
	0.5 + 0.75	33.3 ± 14.9 ^cde^	0.9 ± 0.3 ^ab^
	0.75 + 0.5	53.3 ± 16.9 ^abc^	1.1 ± 0.3 ^ab^
	1.0 + 0.1	86.6 ± 8.1 ^a^	1.7 ± 1.7 ^a^
TDZ	0.5	13.3 ± 8.1 ^de^	0.5 ± 0.3 ^bc^
	0.75	73.3 ± 27.8 ^ab^	1.3 ± 0.2 ^ab^
	1	46.6 ± 16.9 ^bcd^	0.9 ± 0.2 ^ab^

Mean values followed by the same superscripts within a column are not significantly different according to DMRT at *p* ≤ 0.05 level.

**Table 2:** Elemental composition of anther and anther-derived callus of *C. roseus* using SEM–EDX analysis.

S.No.	Element	Anther Explant		Anther-Derived Callus	
		Weight %	Atomic %	Weight %	Atomic %
1	Carbon	33.59	70.67	47.34	79.42
2	Oxygen	12.65	19.97	11.55	14.55
3	Sodium	1.93	2.12	1.63	1.42
4	Phosphorous	0.87	0.71	1.03	0.67

**Table 3:** List of phytocompounds identified in the methanolic extract of field-grown anther of *C. roseus* using GC–MS analysis.

S.No.	RT (min)	Peak Area %	Name of the Compound	Molecular Formula	Molecular Weight
1	3.760	1.62	Ethylcyclopentenolone	C_7_H_10_O_2_	126
2	4.436	1.12	Pyranone	C_6_H_8_O_4_	144
3	5.484	1.17	Coumaran	C_8_H_8_O	120
4	5.739	0.42	1-monoacetin	C_5_H_10_O_4_	134
5	6.287	0.26	6-oxoheptanoic acid	C_7_H_12_O_3_	144
6	6.504	0.38	Indole	C_8_H_7_N	117
7	6.628	0.14	4-vinylguaiacol	C_9_H_10_O_2_	150
8	7.616	2.12	1,2-octanediol	C_8_H_18_O_2_	146
9	9.101	18.42	Guanosine	C_10_H_13_N_5_O_5_	283
10	9.816	0.45	2,6-dimethoxy-4-vinylphenol	C_10_H_12_O_3_	180
11	10.086	0.78	1,2-benzenedicarboxylic acid, diethyl ester	C_12_H_14_O_4_	222
12	10.473	0.09	Cedrol	C_15_H_26_O	222
13	10.796	0.17	Dihydromethyljasmonate	C_13_H_22_O_3_	226
14	11.050	3.78	Quinic acid	C_7_H_12_O_6_	192
15	11.914	0.08	2-benzylideneoctanal	C_15_H_20_O	216
16	12.061	2.86	Mome inositol	C_7_H_14_O_6_	194
17	13.082	0.13	Diisobutyl phthalate	C_16_H_22_O_4_	278
18	13.411	0.11	Heptadecane	C_17_H_36_	240
19	13.681	0.09	Methyl palmitate	C_17_H_34_O_2_	270
20	14.117	0.18	n-hexadecanoic acid	C_16_H_32_O_2_	256
21	14.403	0.12	Eicosane	C_20_H_42_	282
22	15.355	4.71	Hexacosane	C_26_H_54_	366
23	15.883	0.09	Docosanoic acid	C_22_H_44_O_2_	340
24	16.259	0.79	Tetracosane	C_24_H_50_	338
25	16.910	0.35	9-tricosanol acetate	C_25_H_50_O_2_	382
26	17.134	9.78	Hexatriacontane	C_36_H_74_	506
27	17.293	0.20	4,5-dihydro-2-[(8Z,11Z)-8,11-heptadecadienyl]oxazole	C_20_H_35_NO	305
28	17.398	0.19	4,8-cyclododecadien-1-one	C_12_H_18_O	178
29	17.963	1.13	Dotriacontane	C_32_H_66_	450
30	18.571	0.30	Octacosanol	C_28_H_58_O	410
31	18.765	2.82	n-tetracontane	C_40_H_82_	562
32	18.953	0.80	alpha-monostearin	C_21_H_42_O_4_	358
33	19.536	0.22	1-bromotriacontane	C_30_H_61_Br	500
34	20.322	0.11	Linoleyl acetate	C_20_H_36_O_2_	308
35	20.523	0.12	(-)-Coronaridine	C_21_H_26_N_2_O_2_	338
36	21.137	27.01	Squalene	C_30_H_50_	410
37	22.759	0.19	Arachidic acid, 3-methylbutyl ester	C_25_H_50_O_2_	382
38	22.896	0.57	beta-tocopherol	C_28_H_48_O_2_	416
39	23.543	0.30	Vitamin E	C_29_H_50_O_2_	430
40	24.640	1.20	Campesterol	C_28_H_48_O	400
41	24.766	0.30	Ergostan-3-ol	C_28_H_50_O	402
42	25.105	0.08	Trans-24-ethylidenecholesterol	C_29_H_48_O	412
43	25.178	0.52	3-oxocholestane	C_27_H_46_O	386
44	25.440	0.22	p-coumaric acid, 2-methylpropyl ether, 2-methylpropyl ester	C_17_H_24_O_3_	276
45	25.603	2.87	gamma-sitosterol	C_29_H_50_O	414
46	25.762	0.57	Stigmastanol	C_29_H_52_O	416
47	26.055	0.16	Ergosta-4,24(28)-dien-3-one	C_28_H_44_O	396
48	26.132	0.87	4-campestene-3-one	C_28_H_46_O	398
49	26.230	0.23	Cholestanone	C_27_H_46_O	386
50	27.321	2.72	Methyl commate C	C_31_H_50_O_4_	486
51	28.021	5.54	alpha amyrin	C_30_H_50_O	426

**Table 4:** List of phytocompounds identified in the methanolic extract of anther-derived callus of *C. roseus* using GC–MS analysis.

S.No.	RT (min)	Peak Area %	Name of the Compound	Molecular Formula	Molecular Weight
1	3.598	0.58	1,3,5-triazine-2,4,6-triamine	C_3_H_6_N_6_	126
2	4.320	0.10	Isopropylmethylnitrosamine	C_4_H_10_N_2_O	102
3	4.498	5.49	1,2,3-propanetriol	C_3_H_8_O_3_	92
4	5.040	0.23	3-cis-methoxy-5-trans-methyl-1R-cyclohexanol	C_8_H_16_O_2_	144
5	5.270	0.35	Catechol	C_6_H_6_O_2_	110
6	5.402	0.50	2,5,5-trimethylhepta-2,6-dien-4-ol	C_10_H_18_O	154
7	5.508	3.89	5-hydroxymethylfurfural	C_6_H_6_O_3_	126
8	5.735	1.06	1-monoacetin	C_5_H_10_O_4_	134
9	5.949	0.15	Decanoic acid	C_10_H_20_O_2_	172
10	6.304	0.40	4-oxopentyl acetate	C_7_H_12_O_3_	144
11	7.133	0.24	Eugenol acetate	C_12_H_14_O_3_	206
12	8.022	0.07	Indan-1,3-diol monoacetate	C_11_H_12_O_3_	192
13	8.728	6.16	Guanosine	C_10_H_13_N_5_O_5_	283
14	9.764	0.08	Dodecanoic acid	C_12_H_24_O_2_	200
15	10.784	0.15	Dihydromethyljasmonate	C_13_H_22_O_3_	226
16	10.986	0.10	1-(4-isopropylphenyl)-2-methylpropyl acetate	C_15_H_22_O_2_	234
17	11.145	0.27	Benzoic acid, 2-hydroxy-, heptyl ester	C_14_H_20_O_3_	236
18	11.555	0.19	Methyl myristate	C_15_H_30_O_2_	242
19	11.934	0.61	4-((1E)-3-hydroxy-1-propenyl)-2-methoxyphenol	C_10_H_12_O_3_	180
20	12.030	0.11	Tridecanoic acid	C_13_H_26_O_2_	214
21	12.246	0.20	Stearic acid methyl ester	C_19_H_38_O_2_	298
22	12.334	0.72	Octadecanoic acid, methyl ester	C_19_H_38_O_2_	298
23	12.640	0.14	Pentadecanoic acid, methyl ester	C_16_H_32_O_2_	256
24	13.075	0.29	Diisobutyl phthalate	C_16_H_22_O_4_	278
25	13.255	0.03	1-hexadecanol	C_16_H_34_O	242
26	13.298	0.62	Hexadecanoic acid, methyl ester	C_17_H_34_O_2_	270
27	13.467	0.19	Methyl palmitoleate	C_17_H_32_O_2_	268
28	13.580	0.03	7,9-di-tert-butyl-1-oxaspiro(4,5)deca-6,9-diene-2,8-dione	C_17_H_24_O_3_	276
29	13.676	3.96	Methyl palmitate	C_17_H_34_O_2_	270
30	14.113	0.21	n-hexadecanoic acid	C_16_H_32_O_2_	256
31	14.305	0.49	Decyl hexofuranoside	C_16_H_32_O_6_	320
32	14.387	0.50	Eicosanoic acid, methyl ester	C_21_H_42_O_2_	326
33	14.533	0.36	Cis-sinapyl alcohol	C_11_H_14_O_4_	210
34	14.664	0.15	Heptadecanoic acid, methyl ester	C_18_H_36_O_2_	284
35	14.925	0.12	Oxybenzone	C_14_H_12_O_3_	228
36	15.316	3.31	Linoleic acid, methyl ester	C_19_H_34_O_2_	294
37	15.374	1.97	Ethyl oleate	C_20_H_38_O_2_	310
38	15.423	0.72	Oleic acid, methyl ester	C_19_H_36_O_2_	296
39	15.607	0.79	Octadecanoic acid, methyl ester	C_19_H_38_O_2_	298
40	16.399	0.70	cis-10-nonadecenoic acid, methyl ester	C_20_H_38_O_2_	310
41	16.816	0.08	4,8,13-duvatriene-1,3-diol	C_20_H_34_O_2_	306
42	17.290	0.08	4,5-dihydro-2-[(8Z,11Z)-8,11-heptadecadienyl]oxazole	C_20_H_35_NO	305
43	17.335	0.03	(Z)-2-(pentadec-8-en-1-yl)-4,5-dihydrooxazole	C_18_H_33_NO	279
44	17.379	0.16	Methyl arachidate	C_21_H_42_O_2_	326
45	17.589	0.45	6-methyladenine, TMS derivative	C_9_H_15_N_5_Si	221
46	17.888	0.09	Octadecanoic acid, 2,3-dihydroxypropyl ester	C_21_H_42_O_4_	358
47	18.239	0.15	Henicosanal	C_21_H_42_O	310
48	18.746	0.12	Nonadecylpentafluoropropionate	C_22_H_39_F_5_O_2_	430
49	18.948	0.21	alpha-monostearin	C_21_H_42_O_4_	358
50	19.006	0.28	Docosanoic acid, methyl ester	C_23_H_46_O_2_	354
51	19.522	0.21	Vindolinine	C_21_H_24_N_2_O_2_	336
52	19.775	0.12	Methyl tricosanoate	C_24_H_48_O_2_	368
53	20.046	0.09	Octocrylene	C_24_H_27_NO_2_	361
54	20.317	0.25	n-propyl linoleate	C_21_H_38_O_2_	322
55	20.593	0.10	Pleiocarpamine	C_20_H_22_N_2_O_2_	322
56	21.122	0.89	Squalene	C_30_H_50_	410
57	22.404	0.36	(+)-Pericyclivine	C_20_H_22_N_2_O_2_	322
58	22.793	0.47	Ajmalicine	C_21_H_24_N_2_O_3_	352
59	23.162	0.20	Cholesta-4,6-dien-3-ol	C_27_H_44_O	384
60	23.470	0.24	Ajmalicine oxindole	C_21_H_24_N_2_O_4_	368
61	24.641	1.69	Campesterol	C_28_H_48_O	400
62	24.901	1.23	Stigmasta-5,20(22)-dien-3-ol	C_29_H_48_O	412
63	25.035	0.84	19-epiajmalicine	C_21_H_24_N_2_O_3_	352
64	25.187	5.32	3-oxocholestane	C_27_H_46_O	386
65	25.476	2.09	beta-stigmasterol	C_29_H_48_O	412
66	25.600	2.42	gamma-sitosterol	C_29_H_50_O	414
67	25.790	1.76	(E)-1-(6,10-dimethylundec-5-en-2-yl)-4-methylbenzene	C_20_H_32_	272
68	25.990	0.30	(22E)-ergosta-4,7,22-trien-3-one	C_28_H_42_O	394
69	26.137	4.88	4-campestene-3-one	C_28_H_46_O	398
70	26.235	4.62	Cholestanone	C_27_H_46_O	386
71	26.459	4.47	Stigmasterone	C_29_H_46_O	410
72	26.547	0.22	6-dehydroprogesterone	C_21_H_28_O_2_	312
73	26.640	0.56	Cycloartenol	C_30_H_50_O	426
74	26.776	0.20	3,5-cholestadien-7-one	C_27_H_42_O	382
75	26.869	0.61	Ergosta-4,6,22-trien-3-one	C_28_H_42_O	394
76	27.336	7.08	gamma-sitostenone	C_29_H_48_O	412
77	27.448	1.97	24-methylenecycloartanol	C_31_H_52_O	440
78	27.806	0.93	Stigmasta-3,5-dien-7-one	C_29_H_46_O	410
79	28.442	5.87	4,4-dimethylcholestan-3-one	C_29_H_50_O	414
80	28.846	3.78	(22E)-4-methylstigmast-22-en-3-one	C_30_H_50_O	426
81	30.011	5.55	3-acetylcholestan-2-one	C_29_H_48_O_2_	428

**Table 5:** List of important phytocompounds identified exclusively in the methanolic extract of anther-derived callus of *C. roseus* using GC–MS analysis.

S.No.	RT (min)	Name of the Compound	Molecular Formula
1	7.133	Eugenol acetate	C_12_H_14_O_3_
2	12.246	Stearic acid methyl ester	C_19_H_38_O_2_
3	14.533	Cis-sinapyl alcohol	C_11_H_14_O_4_
4	14.925	Oxybenzone	C_14_H_12_O_3_
5	15.316	Linoleic acid, methyl ester	C_19_H_34_O_2_
6	15.423	Oleic acid, methyl ester	C_19_H_36_O_2_
7	19.522	Vindolinine	C_21_H_24_N_2_O_2_
8	20.046	Octocrylene	C_24_H_27_NO_2_
9	20.593	Pleiocarpamine	C_20_H_22_N_2_O_2_
10	22.404	(+)-Pericyclivine	C_20_H_22_N_2_O_2_
11	22.793	Ajmalicine	C_21_H_24_N_2_O_3_
12	25.035	19-epiajmalicine	C_21_H_24_N_2_O_3_
13	25.476	beta-stigmasterol	C_29_H_48_O
14	26.459	Stigmasterone	C_29_H_46_O
15	26.547	6-dehydroprogesterone	C_21_H_28_O_2_
16	26.640	Cycloartenol	C_30_H_50_O
17	27.336	gamma-sitostenone	C_29_H_48_O

**Table 6:** Estimation of nuclear DNA content, genome size and DNA index of anther-derived callus with respect to donor plant of *C. roseus* using flow cytometry technique.

Plant Sample Type	Nuclear DNA Content (pg)	Genome Size (Mbp) *	DNA Index (DI) **
Standard (leaves)	1.51	1476.7	-
Anther-derived callus	0.76	743.2	0.51

* 1 pg = 978 Mbp [18]. ** DNA Index = sample DNA content/standard DNA content.

## Data Availability

All data are presented in the article.

## Supplementary Materials

The following supporting information can be downloaded at: https://www.mdpi.com/article/10.3390/plants12112186/s1, Figure S1A,B: Mass spectra of identified compounds from methanolic extract of anthers and anther derived callus of *C. roseus*.

## Abbreviations

PGRs	Plant Growth Regulators
MS	Murashige and Skoog
NAA	α-Naphthaleneacetic acid
Kn	Kinetin
TDZ	Thidiazuron
DH	Double Haploid
SEM–EDX	Scanning Electron Microscopy–Energy-Dispersive X-ray
FCM	Flow Cytometric Method
DI	DNA Index
GC–MS	Gas Chromatography–Mass Spectrometry
DMRT	Duncan’s Multiple Range Test
