# Supplementary material for: Phytochemical Composition and Detection of Novel Bioactives in Anther Callus of Catharanthus roseus L
Source: Plants (Basel). 2023 May 31;12(11):2186. doi: 10.3390/plants12112186 (PMC10255809; doi:10.3390/plants12112186)
Supplement: Supplementary file 1 [file plants-12-02186-s001.zip › plants-2421377-supplementary.pdf]

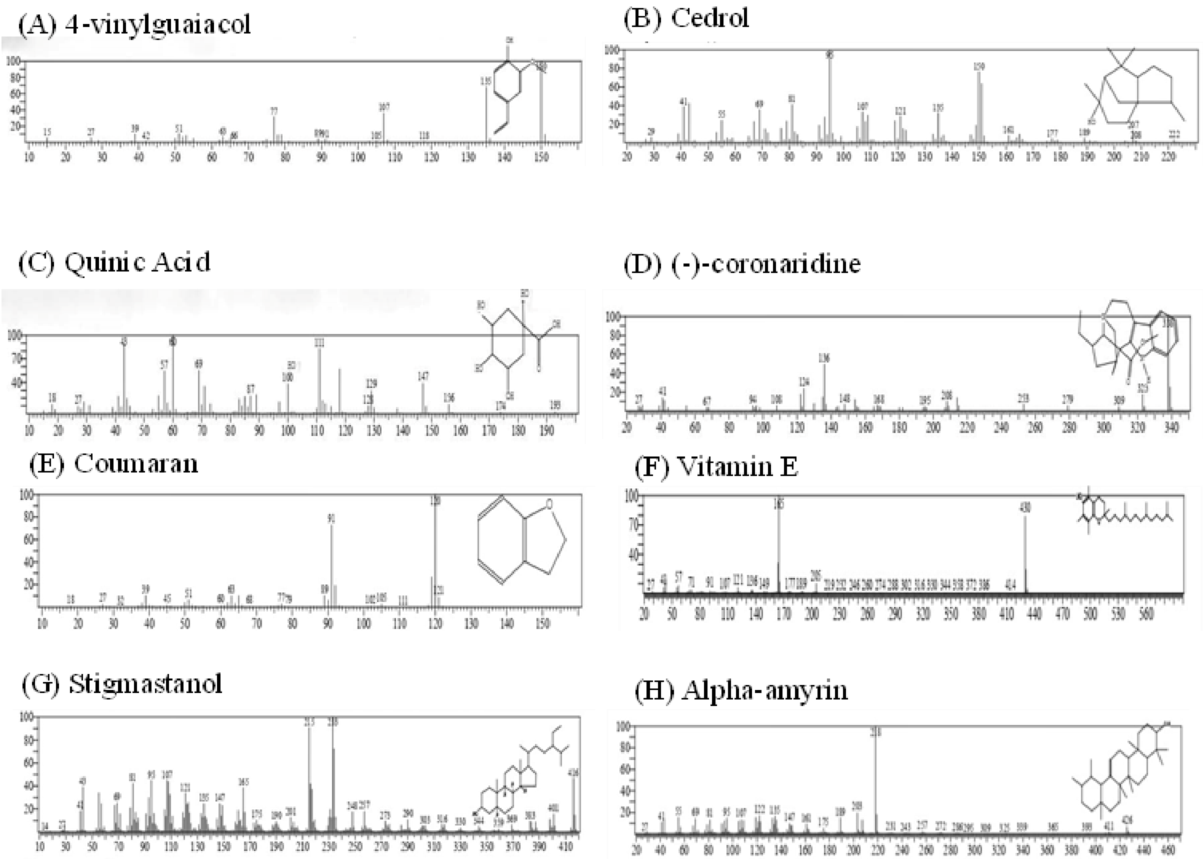

Figure S1. A. Mass spectra of identified compounds from methanolic extract of anthers of C. roseus.

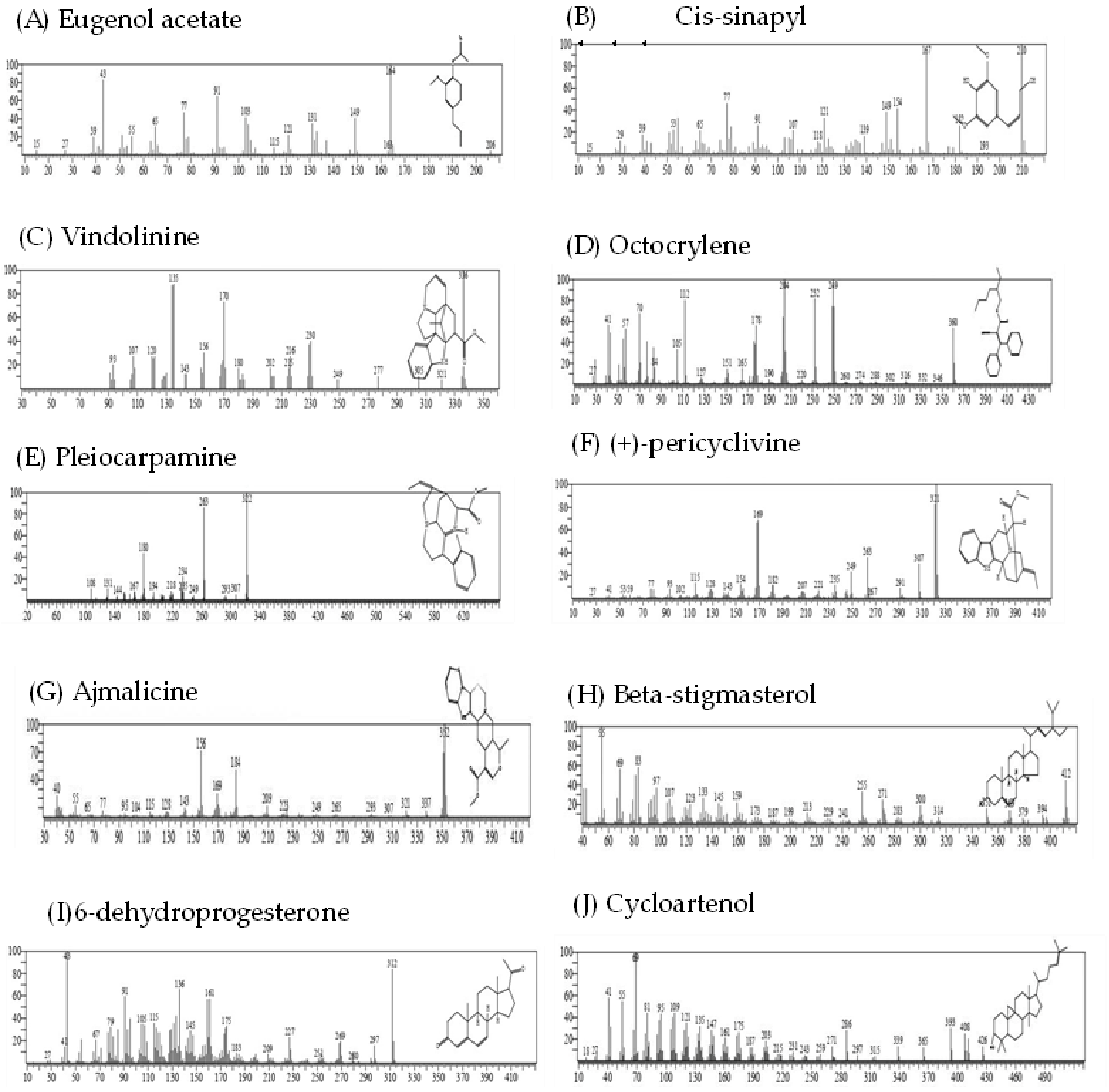

Figure S1. B. Mass spectra of identified compounds from methanolic extract of anther derived callus of *C. roseus*.
